# Is driver education contributing towards road safety? a systematic review of systematic reviews

**DOI:** 10.5249/jivr.v13i1.1592

**Published:** 2021-01

**Authors:** Maryam Akbari, Kamran B. Lankarani, Seyed Taghi Heydari, Seyed Abbas Motevalian, Reza Tabrizi, Mark J.M.Sullman

**Affiliations:** ^*a*^ Health Policy Research Center, Institute of Health, Shiraz University of Medical Sciences, Shiraz, Iran.; ^*b*^ School of Public Health, Iran University of Medical Sciences, Tehran, Iran.; ^*c*^ Non-Communicable Diseases Research Center, Fasa University of Medical Sciences, Fasa, Iran.; ^*d*^ Department of Social Sciences, University of Nicosia, Cyprus.

**Keywords:** Driving education, Road safety, Overview of systematic reviews

## Abstract

**Background:**

There is a vast amount of literature on the effects of driver education. However, the evidence has become somewhat fragmented, making it challenging to understand driver education's effec-tiveness for improving road safety. The current study aimed to provide the efficacy of pre-LDE and post-LDE interventions aimed at improving the safety of drivers (includes crashes, injuries, or secondary outcomes).

**Methods:**

The following online databases were searched up to the 21st of February 2020: Web of Science (WOS), Scopus, PubMed, Cochrane library, and other relevant databases. Systematic reviews (SR) and meta-analyses were selected to investigate the efficacy of driving education in reducing crashes, injuries, or secondary outcomes. Two investigators independently conducted the data extraction and used the assessment of multiple systematic reviews (AMSTAR) tool to conduct a quality assessment of each SR identified.

**Results:**

Out of the 229 potential articles, seven SRs were eligible for the current overview of systematic reviews and meta-analyses. This overview showed that pre-and post-license education by people of all ages led to improvements in secondary outcomes, such as performance, self-perceived driving abilities, behind-the-wheel driving performance, and even a small decrease in traffic offenses. However, education was not effective in reducing crashes or injuries, either at the individual or community level.

**Conclusions:**

There was no evidence that driver education is an effective approach to reducing crashes or injuries. This negative result might be due to ineffective teaching methods. To improve road safety, it appears necessary to change the method or content of driving education since the current approaches to driving education do not reduce traffic crashes or injuries.

## The Problem 

According to a recent road safety report, the aim of reducing road deaths by 50% by 2020 is that in the context of the United Nations Decade of Action for Road Safety, it will not be met.^1^ This report also noted that the progress in reducing average road traffic fatalities in the period 2013-17 has been slower than in the period 2010-13 (0.5% vs. 2.6%).^1^

Internationally, approaches to road safety management have focused on the central pillars, such as engineering, enforcement, education, evaluation, the speed of emergency responses, as well as improving vehicles and roads.^2-3^ Due to the practical and feasibility of training or education, among these central pillars, this is a common approach for improving safety, especially in developing countries that have limited resources.^4^

The training and education of drivers can be placed into two main categories, which are pre-license education and post-license driver education (pre-LDE and post-LDE). Pre-LDE comes in two common forms, school-based road safety education and one-to-one driving instruction with professional driving instructors.^5^ The procedural and cognitive skills training tend to be combined in pre-LDE but are treated separately in post-LDE.^5^ Previous research has found school-based driver education (pre-LDE) has been found to lead to earlier licensing but not to reduce crash involvement among teenagers.^6^ Furthermore, pre-LDE using driving simulators was not found to change the driving style significantly.^7^

Research has found moderately improved knowledge, driving awareness, and driving performance in a number of systematic reviews (SRs) on post-LDE among older people, but no reduction in crashes and injuries.^8-9^ In addition, there is no evidence that education for improving driver behavior and post-LDE has significantly reduced road injuries or crashes, although a small decrease in the occurrence of traffic offenses was reported.^10^ In contrast, there is some evidence that education has a somewhat positive impact on outcomes such as crash rates. However, these studies would be considered low on Sackett's levels of evidence.^11^ In a study,^12^ cognitive training for drivers was found to significantly reduce motor vehicle collision involvement per year and per mile driven. In a group-based education program have been found a reduction in collisions among old-aged drivers by following exposure to a 1 × 90 min session every two years.^13^ Furthermore, a systematic review reported that older driver training, with programs tailored to individual participants, improved their self-perceived driving ability, their behind-the-wheel performance, and reduced crashes.^14^ Therefore, the contribution of driver education towards road safety remains somewhat controversial.

Although there are several systematic reviews (SRs), there are currently no overviews of the systematic reviews and meta-analyses on the efficacy of pre-LDE and post-LDE interventions aimed at improving the safety of drivers (includes crashes, injuries, or secondary outcomes). The current study was conducted to provide a comprehensive overview of pre-LDE and post-LDE efficacy by summarizing the findings from published SRs using data from different countries. And it will answer the question of whether driver training contributes to road safety.

## Methods 

**Search Strategy and Inclusion Criteria**

The methodology of this study is based on the preferred reporting items for systematic reviews and meta-analysis (PRISMA) checklist. We searched online databases, including the Web of Science (WOS), Scopus, PubMed, Cochrane library, and other relevant databases. Searches were performed up to the 21st of February 2020. This study is a systematic review of systematic reviews (SRs). In order to identify SRs that assessed the impact of driver education on crashes, injuries, or secondary outcomes, the following search terms were used: (driver* OR driving) AND (driver training* OR pre-driver training* OR learning to drive training* OR post-licensure training* OR driver improvement * OR novice drivers) AND (young* OR youth* OR teen* OR adolescent* OR novice* OR older* OR student) AND (systematic review* OR meta-analysis*). All identified studies were included in the software StArt (State of the Art through Systematic Review).^15^ This software used different combinations of the terms "systematic" and "review" in the title or abstract to identify the other studies' systematic reviews. These studies were also screened separately by two other individuals in addition to the software.

SR studies were included that met the following criteria: studies were systematic reviews with or without a meta-analysis that covered the effect of driver education [included pre-license driver education (professional driving instruction, school-based driver education, and simulator training) and post-license driver education (novice drivers education, remedial driver education, advanced driver education, and driver improvement)] on road traffic outcomes [(crash and injury) and secondary outcomes (driving performance or driving awareness or driving behavior and knowledge)].

**Data Extraction and Quality Assessment**

Two investigators independently conducted the data extraction and quality assessment of all SRs include in this study. Discrepancies were resolved by consensus between the two or by discussions with a third researcher (K-BL). 

The following data were extracted from all SRs included: first author’s name, publication year, study country, number of included primary studies, type of included primary studies, the stage of the driver, education type, age group, whether they studied crashes, injuries or secondary outcomes, and their main findings. The assessment of multiple systematic reviews (AMSTAR) tool was used to assess all systematic reviews' methodological quality.^16^ This checklist assessed the quality of the SRs according to the following items: ‘a priori’ design provided; duplicate study selection/data extraction; comprehensive literature search; status of publication (i.e., grey or unpublished literature); list of studies included/excluded provided; characteristics of included studies documented; scientific quality assessed and documented; appropriate formulation of conclusions (based on methodological rigor and scientific quality of the studies); appropriate methods of combining studies (homogeneity test, effect model used, and sensitivity analysis); assessment of publication bias (graphic and/or statistical test); and conflict of interest statement.

## Results

**Characteristics of Included Studies**

**Figure 1:**
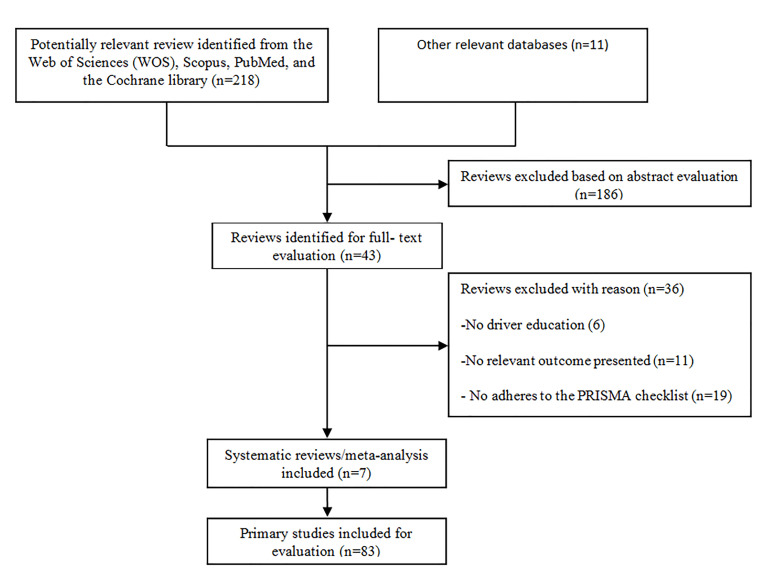
Flowchart of study identification and selection process.

Our search strategy identified 229 articles, of which 49 were duplicates and were therefore removed. After title and abstract screening, 43 records were found to be eligible for full-text assessment (Appendix 1), but only seven were found to meet all of these overview criteria. The flowchart of the study identification and selection process is presented in Figure 1.

**Appendix 1:** Reviews identified for full-text evaluation

Title	Authors	Reason of excluded
Is there a case for driver training? A review of the efficacy of pre- and post-licence driver training	Vanessa Beanland et al.^5^	No adherence to the PRISMA check-list(no systematic review)
Efficacy of training with driving simulators in improving safety in young novice or learner drivers: A systematic review	Luis Miguel Martín-delosReyes et al.^7^	No relevant outcome presented
Post-licence driver education for the prevention of road traffic crashes: a systematic review of randomised controlled trials	Katharine Ker & et al.^10^	No relevant outcome presented
The effectiveness of road safety education	Nina Dragutinovic et al.^18^	No driver education
Education in road safety-are we getting it right?	Frank McKenna et al.^19^	No adherence to the PRISMA check-list(no systematic review)
The effectiveness of driver training as a road safety measure: A review of the literature	Christie, Ron et al.^24^	No adherence to the PRISMA check-list(no systematic review)
Scoping Review of the Driving Behaviour of and Driver Training Programs for People on the Autism Spectrum	Nathan J. Wilson et al.^30^	No adherence to the PRISMA check-list(no systematic review)
The efficacy of advanced driver training: A targeted literature review	Vanessa Beanland et al.^31^	No adherence to the PRISMA check-list(no systematic review)
Do driver training programs reduce crashes and traffic viola-tions? A critical examination of the literature	Raymond C. Peck et al.^32^	No adherence to the PRISMA check-list(no systematic review)
European advanced driver training programs: Reasons for opti-mism	Simon Washington et al.^33^	No adherence to the PRISMA check-list(no systematic review)
Assessment. Driver education and training in post-primary schools	Ray Fuller et al.^34^	No adherence to the PRISMA check-list(no systematic review)
Good Practice in Pre-driver education	Carole Deighton et al.^35^	No adherence to the PRISMA check-list(no systematic review)
The Effectiveness of driver Education Programs in Reducing Traffic Accidents in Saudi Arabia	Al-Subhi, Suhail S et al.^36^	No adherence to the PRISMA check-list(no systematic review)
Simulators, driver education and disadvantaged groups: A scop-ing review	Lyndel Bates et al.^37^	No relevant outcome presented
A comparison of driver education effectiveness: summer programs versus semester-long programs	Gary E. Anker et al.^38^	No adherence to the PRISMA check-list(no systematic review)
Education, publicity and training in road safety: A litera-ture review	Michael Henderson et al.^39^	No adherence to the PRISMA check-list(no systematic review)
Road Safety Education and Training from a Public Health Perspective	Ron Christie et al.^40^	No relevant outcome presented
Effectiveness and Role of Driver Education and Training in a Graduated Licensing system	Mayhew, Daniel R et al.^41^	No adherence to the PRISMA check-list(no systematic review)
What is the Effect of Driver Education Programs on Traffic Crash and Violation Rates?	Stephen Michael et al.^42^	No relevant outcome presented
Evaluation of pre-driver education program	Haworth Narelle et al.^43^	No adherence to the PRISMA check-list(no systematic review)
The safety value of driver education an training	D R Mayhew et al.^44^	No relevant outcome presented
An examination of what the currently available data can tell us about the effects on offence and crash history of two driver education programs.	Ben Lewis-Evans et al.^45^	No relevant outcome presented
The Effectiveness of Road Hazard Perception Training: Litera-ture Review	Justina Slavinskienė et al.^46^	No driver education
A review of educational and legislative strategies to promote bicycle helmets	Philip L Graitcer et al.^47^	No driver education
Large-Scale Evaluation of Driver Education Review of the Literature on Driver Education Evaluation 2010 Update	Lawrence Lonero et al.^48^	No relevant outcome presented
Review of the ACT Road Ready and Road Ready Plus Novice Driver Road Safety Education Course Material	Alexia Lennon et al.^49^	No adherence to the PRISMA checklist(no systematic review)
Trends in Driver Education and Training	Lawrence P. Lonero et al.^50^	No relevant outcome presented
Young novice drivers, driver education and training: Literature review	Inger Engström et al.^51^	No adherence to the PRISMA checklist(no systematic review)
The effectiveness of driver training as a road safety measure	Christie, Ron et al.^52^	No relevant outcome presented
The Effectiveness of Driver Training/Education as a Road Safety Measure	Christie, Ron et al.^53^	No adherence to the PRISMA checklist(no systematic review)
Graduated driver licensing: An international review	Lyndel J. Bates et al.^54^	No relevant outcome presented
The Roles and Performance of Professional Driving Instructors in Novice Driver Education	Zulhaidi M. Jawi et al.^55^	No adherence to the PRISMA checklist(no systematic review)
Is evidence in practice? Review of driver and cyclist education materials with respect to cycling safety evidence	Meghan Winters et al.^56^	No adherence to the PRISMA checklist(no systematic review)
Safety education of pedestrians for injury prevention: a systematic review of randomised controlled trials	Olivier Duperrex et al.^57^	No driver education
The efficacy of road safety education in schools: A review of current approaches	SJ Raftery et al.^58^	No driver education
Child–parent interaction in relation to road safety education: part 1–A critical literature review	Mima Cattan et al.^59^	No driver education

Five of the included studies were systematic reviews,^7-9.14,17^ with two of them also including a meta-analysis.^6,10^ There were 83 primary driver education interventions included in this current overview, five of which were conducted on post-license education^8-10,14,17^ and two on pre-license education.^6,7^ The publication years of the SRs included ranged from 1999 to 2019. Table 1 presents the detailed characteristics of the included SRs.

**Table 1:** Summary of driver education evaluation result.

Reference	Countries	Study design	Number of Studies included	Type of studies	Driver stages	Type of education	Age group	Outcomes	Summary of findings
Kua et al. (2007)^9^	Canada	Systematic review	8	Six RCTs, one pre-post-study design,and one descriptive study	Post-license	Educational curriculum (retraining)	aged 55 and older	Awareness, driving behavior, crashes	Training interventions in older drivers improve moderately driving awareness and driving behavior, but do not reduce crashes.
Vernick et al. (1999)^17^	USA	Systematic review	9	Two randomized controlled trials,9,10 two re-analyses of the data from one of the controlledtrials,11,12 three ecologic time-series designs,13–15 and two ecologic analyses of group differences	Post-license	Education curriculum	High School (young drivers)	Motor vehicle crash	there is no convincing evidence among young drivers that the driver education of high school reduces motor vehicle accident involvement rates (either at the community or individual level)
Ker et al. (2005)^10^	UK	Systematic review and meta-analysis	21	RCT	Driver improvement /Post-license	Remedial driver education / Advanced driver education	All ages	Re-offences , crashes, injury	No evidence of that post-licence driver training is effective in reducing road crashes (relative risk (RR): 0.98, 95%CI: 0.96-1.01) or injury crashes (RR: 1.12, 95%CI: 0.88-1.41); however the results showed a small reduction in the traffic offences (RR: 0.96, 95%CI: 0.94-0.98).
Roberts et al. (2008)^6^	UK	Systematic review and meta-analysis	3	RCT	Pre-license	School based driver education	Aged 17 to 21 years	Crashes	No evidence of that the training of drivers reduce road crashes (Risk Ratio: 1.03 95%CI: 0.98-1.08).
Korner-Bitensky et al. (2009)^8^	Canada	Systematic review	4	Three RCTs and one matched-pairs cohort design	Post - license	Education modules, educational curriculum	Aged 55 and older	Knowledge, driving performance, crashes	Combined education with on-road training strongly improves the driving performance and moderately increases the driving knowledge. Physical restraining moderately improves the performance of drivers. There is moderate evidence that a single training intervention curriculum is not efficacy in decreasing crashes
Sangrar et al. (2019)^14^	Canada	Systematic review	33	Twenty-five RCTs and eight non-RCTs	Post - license	Classroom-based education, or a combination of classroom-based education with on-road feedback	Aged 55 and older	Knowledge, crashes	Increased drivers’ knowledge about road safety. And improved the self-perceived and behind-the-wheel outcomes such as crashes.
Martín-delosReyes et al (2019)^7^	Spain	Systematic review	5	Two experimental studies with random assignment, one quasi-experiment, and two cohort studies	Pre – license (learner drivers)	Driving Simulator-based training	Young novice or learner drivers	Safety	No evidence to support or refute the program education efficacy using simulators among young people in developing the driving behavior and their safety.

**Pre-license Driver Education (Pre-LDE)**

Pre-LDEs are defined as any educational curriculum designed to encourage the development of driving-related behaviors and beliefs that are specifically targeted at those who have not yet received their provisional driver's license.^5^ Pre-LDE includes many different formats, including professional driving instruction, school-based driver education, and simulator training.^5^

A systematic review of three randomized controlled trials (RCTs), conducted by Roberts and Kwan,^6^ found that road safety education in schools contributes to earlier licensing of teenage attendees. Furthermore, they also suggested that these interventions may potentially lead to a significant rise in the proportion of crashes among adolescents who had been through this course, via earlier licensing.

A systematic review conducted by Martín-delosReyes et al.^7^ investigated whether the use of driver simulator training in young learner drivers or inexperienced drivers leads to a reduction in road crash or injury and/or traffic offenses, or to the development of safer driving skills when compared with non-simulator-based training. The results of this systematic review did not support the efficacy of simulator-based training programs for improving the safety of driving styles among young learner or novice drivers.

**Post-license Driver Education (Post-LDE)**

Post-LDEs are focused on inexperienced drivers (mostly young) with the aim of reducing their greater risk of being crash involved.^5^ Although pre-licensing driver training attempts to incorporate cognitive and procedural skills training within a single curriculum, post-licensing driver training programs attempt to address either procedural or cognitive skills.^5^

Vernick et al. ^17^ performed a systematic review of Post-LDEs to investigate whether high school-age individuals participating in driver education had fewer motor vehicles accidents or violations, or were much more likely to obtain a driver's license, compared to those not participating in these programs. They also investigated whether the presence of high school driver training programs was linked to lower rates of motor vehicle crashes among inexperienced drivers. The results of this review of nine studies found that there was no compelling evidence to suggest that high school driver education decreased the number of motor vehicle accidents among teenage drivers, either at the individual or community level. In contrast, there was evidence that these programs were linked with higher rates of involvement in young drivers' crash or injury, as they led to early licensure.

Several systematic reviews among older drivers have indicated that driver training has little or no direct impact on road safety, in terms of crash or injury risk reduction.^8,9,14^ However, there is evidence that these programs can have a beneficial effect on several secondary outcomes (e.g. enhancing driving performance, or driving actions and knowledge or driving awareness). ^8,9,14^ In another systematic review of post-license driver education,^10^ which primarily looking at remedial educational programs, the researchers again concluded that there was no clear evidence that these programs reduce crash or injury, and only very poor evidence that they minimize re-offending.

**Quality Assessment**

The final scoring of the methodological quality of each SR is presented in Table 2. Based on the AMSTAR methodology, the overall score for six SRs was assessed to be high and one was found to be of medium quality.

**Table 2:** Methodological quality assessment of the included systematic reviews.

Authors	1	2	3	4	5	6	7	8	9	10	11	Scoring
**Kua et al. (2007)^9^**	Yes	Yes	Yes	No	No	Yes	Yes	Yes	Yes	Yes	No	High(8)
**Vernick et al. (1999)^17^**	No	Yes	No	No	No	Yes	Yes	Yes	Yes	No	No	Medium (5)
**Ker et al. (2005)^10^**	Yes	Yes	Yes	Yes	No	Yes	Yes	Yes	Yes	Yes	No	High(9)
**Roberts et al. (2008)^6^**	Yes	Yes	Yes	Yes	Yes	Yes	Yes	Yes	Yes	Yes	Yes	High (11)
**Korner-Bitensky et al. (2009)^8^**	Yes	Yes	Yes	Yes	Yes	Yes	Yes	Yes	Yes	No	No	High (9)
**Sangrar et al. (2019)^14^**	Yes	Yes	Yes	Yes	Yes	Yes	Yes	Yes	Yes	Yes	Yes	High (11)
**Martín-delosReyes et al (2019)^7^**	Yes	Yes	Yes	Yes	No	Yes	Yes	Yes	Yes	No	No	High(8)

All 11-items were scored as “Yes”, “No”, “Can’t Answer” or “Not Applicable”. AMSTAR comprises the following items: 1. ‘a priori’ design provided; 2. Duplicate study selection/data extraction; 3. Comprehensive literature search; 4. Status of publication as inclusion criteria (i.e., grey or unpublished literature); 5. List of studies included/excluded provided; 6. Characteristics of included studies documented; 7. Scientific quality assessed and documented; 8. Appropriate formulation of conclusions (based on methodological rigor and scientific quality of the studies); 9. Appropriate methods of combining studies (homogeneity test, effect model used and sensitivity analysis); 10. Assessment of publication bias (graphic and/or statistical test); and 11. Conflict of interest statement.

## Discussion

To the best of our knowledge, this current systematic review of systematic reviews is the first study to investigate the efficacy of pre-LDE and post-LDE aimed at improving the safety of drivers. Based on 7 SRs (5 SRs and 2 Meta). The present study found that pre- and post-license driver training improved driving performance, self-perceived driving ability, and may also result in a small decrease in traffic offenses. However, these educational interventions were not effective in reducing crashes and injuries, either at the individual or community level.

Driver education is a popular approach for improving road safety, ^44^ but our study indicates that driver education has not been effective in achieving its main outcome, which is reducing crashes, injuries, and deaths. This negative finding may be due to the use of ineffective teaching methods, ineffective course content, failure to understand the needs of adult learners, or not targeting the correct risky driving behaviors. 

Educational interventions may not always have the intended effect on all individuals, which needs to be taken into consideration. It has been found that raising awareness of risk may reduce the subject's attractiveness for some learners, while for others it may be more at-tractive and more likely to lead to action.^18^ Furthermore, the systematic review by Roberts et al.^6^ reported that school-based driver education interven-tions can even lead to more harm, by reducing the age at which they obtain a license and thus leading to an increase in the proportion of teenagers involved in traf-fic crashes.

Some researchers have argued that road safety education interventions frequently focus on vehicle control skills, which are not often the most important issue when it comes to crash involvement.^18,19^ Perhaps road safety educations focuses too strongly on a small number of driving behaviors, such as speed and neglect other important behavioral aspects, such as smoking, alcohol, and drug use. In such a case, training recipients, even if they show improvements in their attitudes and driving speed, the negative impact of other behaviors, such as alcohol consumption will continue to increase the number of crashes and their related injuries.^18^ In fact, some educational interventions satisfy a number of goals but do not necessarily target all of the important causes, which reduces the efficiency of such interventions.^19^ Furthermore, most educational interventions only provide general information about safety, without providing drivers a clear understanding of their own behavior as a road user. Many education interventions focus on improving knowledge and attitudes, rather than an individual’s actual behavior, which reduces these interventions' efficiency. ^19^ In other words, drivers may have an awareness of the risks and the appropriate knowledge, but still engage in unsafe behavior even after education program.^18^

The ineffectiveness of education programs may be caused an implementation without appropriate scientific evidence or being based upon educational theory. Some researchers believe that designing an educational intervention without regard to guiding theory is like prescribing a medical drug to a patient regardless of the patient's physiology.^18,20^ Nasvadi and Vavrik suggested that before making decisions regarding driving education, it is important to carefully assess the potential benefits and contents in order to optimize the efficacy of the interventions.^21^ Furthermore, another problem with driver education-based interventions is that designers do not include appropriate behavior change techniques.^22-23^

In the case of safe traffic behavior, it has been argued that the resources allocated to educational programs may fail to improve traffic safety since traffic collisions and their related injuries do not decrease and drain already stretched budgets and hinder the development of more creative and innovative solutions.^24^ The ineffectiveness of training and education programs have also been reported in other areas, prompting the British Medical Association to conclude that many educational interventions are politically attractive, but have been found to be ineffective.^25^

The implementation method of educational programs can also influence its effectiveness. For example, it has been shown that educational activities are often implemented in short time periods.^26^ Short-term education includes the length of the session, the number of sessions, and the interval between sessions. The use of short-term interventions may reduce the long-term effectiveness of education or training for many individuals.^18^ Continuous driver education should be conducted in an evidence-based holistic framework, using road safety interventions such as the enforcement of traffic safety and risk reduction by decreasing and controlling early exposure.^27-28^

Our conclusion should be interpreted with caution on the effects of education on road safety, because first we should be ensured that the evaluation of interventions across primarily included studies has conducted with a scientific method, appropriately.^17^ For example, when the driver education cannot reduce the crash or death rate, this failure may be manifested in the inability of young drivers' function, inexperience, or interaction of other characteristics, instead of reflecting inability education interventions.^21^ Other study by Zhang et al.,^29^ reported that driving educational interventions were effective only among careful drivers. And also, the period between getting safe behavior and reducing road crash and injury in the community is a very long and time-consuming phenomenon. So measuring the rate of road traffic injuries and education's effect seems not to be a rational manner. Instead, the effect of education interventions should be measured on safe traffic related behaviors. On the other hand, when educational interventions accompanied by other important factors such as road safety; vehicle quality and safety; and even signalization, policy, legislation, law enforcement, etc, it could improve safe behavior.^14^

In brief, our findings are important to consider for stakeholders and policymakers. Driving education was not found to be an effective approach for improving the main outcomes, which are crashed and injuries. The reasons for this need to be considered in future research.

This study examines the efficacy of pre-LDE and post-LDE interventions to improve the safety of drivers (includes crashes, injuries, or secondary outcomes). A review of systematic review studies can give us a complete result. One of the strengths of this study was the qualitative review of the studies, most of which were of high quality. Also, a review of several large databases demonstrates the comprehensiveness of this study. However, the limitations of the study should not be overlooked. Among other things, numeric data was not possible to perform meta-analysis and comment in numerical data. Also, there may be overlap between the initial studies included in the systematics understudy and have achieved similar results in all respects.

## Conclusion

There is no evidence the pre-LDE or post-LDE driver education leads to a reduction in crashes or injuries. This negative finding may be due to the use of ineffective teaching methods, the absence of effective techniques for changing attitudes or future behaviors, failure to understand the needs of adult learners, or not targeting the correct risky behaviors. 

**Acknowledgments**

This study was undertaken as part of a Ph.D. thesis and was supported by a grant awarded from the Deputy of Research and Technology at the Shiraz University of Medical Sciences (Grant No.1396-01-104-16210).
